# Severe aquaporin 4-IgG-positive neuromyelitis optica with disseminated herpes zoster in a pregnant woman successfully treated with intravenous immunoglobulin

**DOI:** 10.1177/2055217318758119

**Published:** 2018-03-09

**Authors:** Yuki Matsumoto, Mario Tsuchiya, Shakespear Norshalena, Chikako Kaneko, Jin Kubo, Teiji Yamamoto, Toshiyuki Takahashi, Kazuo Fujihara

**Affiliations:** Department of Neurology, Southern Tohoku General Hospital, Japan; Department of Neurology, Southern Tohoku General Hospital, Japan; Department of Neurology, Southern Tohoku General Hospital, Japan; Department of Neurology, Southern Tohoku General Hospital, Japan; Department of Neurology, Southern Tohoku General Hospital, Japan; Department of Neurology, Southern Tohoku General Hospital, Japan; Department of Neurology, NHO Yonezawa Hospital, Japan; Department of Neurology, Tohoku University Graduate School of Medicine, Japan; Department of Multiple Sclerosis Therapeutics, Fukushima Medical University School of Medicine, and Multiple Sclerosis & Neuromyelitis Optica Center, Southern Tohoku Research Institute for Neuroscience, Japan; Department of Neurology, Tohoku University Graduate School of Medicine, Japan

**Keywords:** Neuromyelitis optica, aquaporin-4-IgG, pregnancy, intravenous immunoglobulin, acute treatment, herpes zoster

## Abstract

A 26-year-old, 17-week pregnant woman developed aquaporin-4-IgG-positive severe longitudinally extensive transverse myelitis during the course of disseminated herpes zoster and became quadriparetic. She was unresponsive to high-dose intravenous methylprednisolone but became able to walk without assistance after intravenous immunoglobulin. One and a half months later, left optic neuritis developed but her vision improved with intravenous immunoglobulin. The only sequela was left T5 girdle sensation, and she delivered a healthy baby. Intravenous immunoglobulin may be a rescue therapy in aquaporin-4-IgG-positive neuromyelitis optica attacks in pregnant women, especially those with severe infections.

## Introduction

Aquaporin-4 (AQP4)-antibody-seropositive neuromyelitis optica (NMO) often affects women in their childbearing years. There have been only a few reports of acute-phase treatment in pregnant patients with seropositive NMO. ^1^ In relapses of seropositive NMO, the first-line therapy is intravenous methylprednisolone (IVMP), and plasma exchange (PLEX) is a rescue therapy in those unresponsive to IVMP.^1^ There are also several reports suggesting therapeutic efficacy of intravenous immunoglobulin (IVIg) in acute-phase NMO,^2–7^ but there are no reports of pregnant patients with seropositive NMO treated with IVIg as a rescue therapy.

Here we describe a pregnant woman who developed seropositive NMO following disseminated herpes zoster. She was refractory to IVMP but successfully treated with IVIg. We obtained the patient’s written consent and the institutional review board’s approval for publication.

## Case report

The patient was a 26-year-old pregnant woman (gravida 3 partus 2). In May 2016, the patient felt burning pain over her neck, both arms and anterior chest. One week later, she had vesicular skin lesions with crusts and erosions suggestive of varicella zoster infection in those areas. After two more weeks, she had left hemiparesis that then progressed to quadriparesis. In her 17th week of pregnancy, the patient was referred to our hospital by a local obstetrician.

On admission, the patient was bedridden and could not even sit up. She had a high fever (38.8°C) and severe tingling pain below the neck level aggravated by minor stimuli, which was compatible with allodynia. She also had constipation lasting a week. Physical examination revealed oral candidiasis and vesicles accompanied by crusts and erosions over her right C4, bilateral C5–6 and left Th3 areas. Neurological examination showed profound quadriparesis (manual muscle strength of Medical Research Council 3/5 over the arms and 2/5 over the legs), generalized hyperreflexia, severe hypoesthesia in legs and bowel-bladder disturbance. Varicella-zoster virus (VZV) polymerase chain reaction and VZV-immunoglobulin M (IgM) were negative in the cerebrospinal fluid collected at the onset of myelitis. The patient also had hyponatremia (120 mEq/l) that was asymptomatic and resolved on day 8.

**Figure 1.**
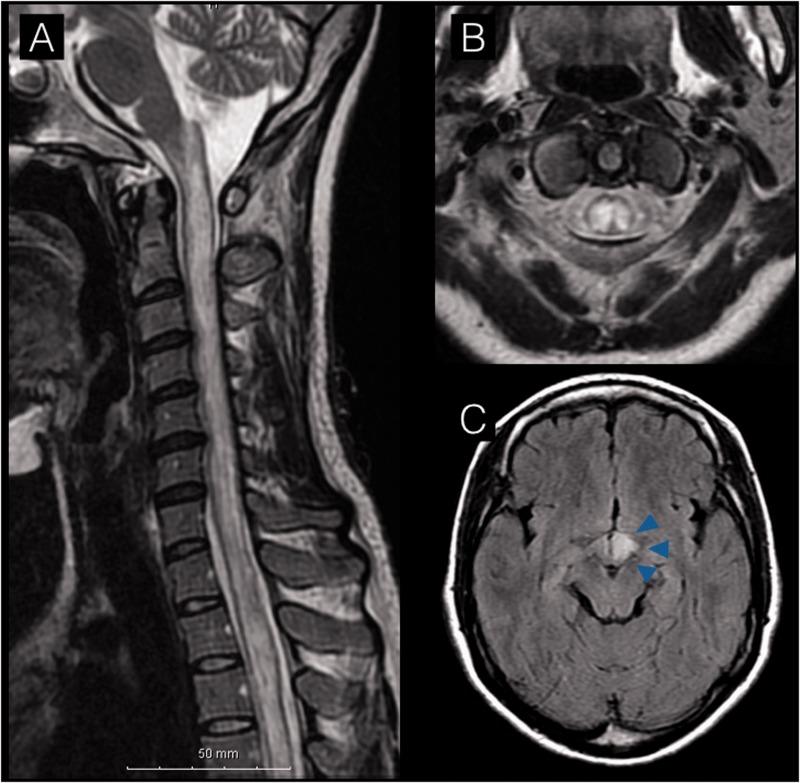
(a) Spinal magnetic resonance imaging on day 2 exhibited a T2-hyperintense lesion extending from the medulla oblongata to the thoracic cord. (b) Axial image at the C1 level revealed an extensive lesion, mainly involving the central gray matter. (c) Axial image at the midbrain level showed a left hypothalamic lesion. (a) and (b): T2-weighted images. (c): Fluid-attenuated inversion recovery image.

Brain and spinal magnetic resonance imaging (MRI) revealed a left hypothalamic T2-hyperintense lesion and a longitudinally extensive transverse lesion extending from the lower medulla down to Th6 (Figure 1). However, she did not have dyspnea, hiccups or nausea/vomiting. The patient was treated with acyclovir (1500 mg/day) and heparin.

We suspected NMO at that point, and administered intravenous methylprednisolone (IVMP) (1 g/day for five days) on the day of admission. However, her symptoms deteriorated and she became completely paraplegic. Then, we treated her with intravenous immunoglobulin (IVIg) (0.4 mg/kg/day) rather than plasma exchange (PLEX) because of risks of suspected sepsis and circulatory instability affecting the fetus.

After IVIg was given on days 6 and 7, she developed a headache, and thus we discontinued the therapy. Although she barely moved her toes on day 7, she became able to raise her right knee on day 10. On day 13, serum AQP4-immunoglobulin G (IgG)) was found to be strongly positive (titers > 75 U/ml), thus fulfilling the diagnostic criteria of NMO with AQP4-IgG.^8^ The patient could not even brush her teeth on day 6 but was able to eat her meals without assistance on day 13. Then she received additional doses of IVIg from day 15 to day 17 with no side effects. Following this, she became able to raise her knees on day 16, dorsiflex her ankles on day 17 and stand up with assistance on day 21. She was given IVIg again from day 25 to day 29 and was able to walk with a walker on day 33. She needed laxatives and disimpaction for constipation and placement of an indwelling urinary catheter for urinary retention until day 29. But urinary and bowel symptoms improved thereafter and herpes zoster was epithelialized and scarred by day 33.

On day 41, she noticed left orbital pain and blurred vision. Her left visual acuity was 20/200 and upper altitudinal hemianopia was observed, indicating the development of left optic neuritis. Then, IVMP (1 g/day for five days) was given but she was refractory to it (20/400 in one week). She was then treated with the same doses of IVIg again but it had to be discontinued because of vomiting two days later. When she was discharged on day 70, she was able to read the headlines of a newspaper with remaining girdle sensation over the left T5 level. A month later, the patient gave birth at 37 weeks’ gestation to a baby without any complications via spontaneous vaginal delivery. Her visual acuity gradually returned to normal although she still had the left T5 girdle sensation.

## Discussion

Rescue therapy for severe NMO attacks refractory to IVMP in pregnant women with concomitant infections is not well established. There have been two cases of acute exacerbations of NMO in pregnancy that failed to respond to IVMP but were successfully treated with PLEX (Table 1 (a)).^1,9^ In our patient with corticosteroid-resistant NMO attacks, we chose IVIg rather than PLEX on the assumption that IVIg might also be effective for infections (disseminated herpes zoster, candidiasis and possible sepsis) as well as NMO. In fact, cases of infectious or parainfectious NMO/longitudinally extensive transverse myelitis (LETM) (mostly associated with herpes zoster) have been reported (Jarius S, et al. J Neurol Sci. 2009) and there are some reports on safety and efficacy of IVIG in immune-mediated diseases with concomitant infections (Simoes J, et al. J Clin Rheumatol. 2015).^10,11^ Possible mechanisms of IVIG efficacy in NMO spectrum disorder may include modulations of AQP4-IgG binding and catabolism, complement activation and B-cell function (Wingerchuk DM. J Clin Immunol 2013). Moreover, IVIg is unlikely to cause circulatory instability adversely affecting the fetus, in contrast to PLEX. Despite short-term IVIg administrations because of adverse effects, the patient experienced significant neurological improvement of LETM while the improvement of optic neuritis was slower. IVIg has also been reported to be safe in neuroimmunological disorders such as myasthenia gravis and pregnancy.^12^

**Table 1.** (a) Case reports of therapeutic plasma exchange in AQP4-IgG-positive NMO during pregnancy and (b) case reports of intravenous immunoglobulin therapies in acute phase of AQP4-IgG-positive NMO.

**Table 1 (a)**	**Age (gestation weeks)**	**Onset or relapse**	**Clinical symptoms and MRI findings**	**Acute treatment before PLEX**	**Times of PLEX**	**Effects of PLEX**	**Reference**
Case 1	25 (17)	Relapse	Respiratory failure, cervical and thoracic LETM	Corticosteroids (unknown details)	Four times	Complete recovery	Cornelio 2009
Case 2	27 (20)	Relapse	Paraplegia, cervical and thoracic LETM	IVMP	Five times	Partial recovery	Rubio Tabares 2016
**Table 1 (b)**	**Age, sex**	**Onset or relapse**	**Clinical symptoms and MRI findings**	**Acute treatment before IVIg**	**IVIg**	**Effects of IVIg**	**Reference**
Case 3	49, F	Onset	Gait disturbance, myelitis from Th4 to Th6	IVMP	0.4 g/kg per day over five days	Complete recovery	Ii 2008
Case 4	81, M	Onset	Paraplegia, myelitis from Th5 to Th11	IVMP	Unknown	Complete recovery	Nakano 2009
		Relapse	Respiratory failure, LETM from medulla oblongata to C6	IVMP and IVIg	Unknown	No improvement	Nakano 2009
Case 5	32, F	Onset	Cognitive impairment, periventricular white matter lesions	IVMP	0.4 g/kg per day over five days	Partial recovery	Stübgen 2012
Case 6	37, M	Onset	Cognitive impairment, periventricular white matter lesions	IVMP	0.4 g/kg per day over five days	Partial recovery	Stübgen 2012
Case 7	28, F	Relapse	ON	IVMP	Unknown	Partial recovery	Romanelli 2014
Case 8	66, F	Onset	ON and myelitis	Corticoids (unknown details)	Unknown	Complete recovery	Hervás-García 2014
Case 9	43, F	Relapse	Respiratory failure, cervical myelitis	None	2 g/kg per day over five days	Partial recovery	Elsone 2014
Case 10	38, F	Relapse	Bilateral ON	PLEX, oral PSL	2 g/kg per day over five days	No improvement	Elsone 2014
Case 11	49, F	Relapse	Paraplegia, thoracic LETM	IVMP	2 g/kg per day over five days	No improvement	Elsone 2014
Case 12	40, F	Relapse	Tetraplegia and heart failure, cervical LETM	IVMP followed by oral PSL	2 g/kg per day over five days	Partial recovery	Elsone 2014
Case 13	79, F	Relapse	Tetraplegia, cervical LETM	IVMP followed by oral PSL	2 g/kg per day over five days	No improvement	Elsone 2014
Case 14	57, M	Relapse	Tetraplegia, cervical and thoracic LETM	IVMP	2 g/kg per day over five days	Improved to baseline function	Elsone 2014
Case 15	38, F	Relapse	Tetraplegia, cervical LETM	IVMP, PLEX followed by oral PSL	2 g/kg per day over five days	Improved to baseline function	Elsone 2014
Case 16	55, M	Relapse	Bilateral ON	Oral PSL	2 g/kg per day over five days	No improvement	Elsone 2014

AQP4: aquaporin 4; F: female; IgG: immunoglobulin G; IVIg: intravenous immunoglobulin; IVMP: intravenous methylprednisolone; LETM: longitudinally extensive transverse myelitis; ON: optic neuritis; M: male; MRI: magnetic resonance imaging; NMO: neuromyelitis optica; PLEX: plasma exchange; PSL: prednisolone.

In NMO, 14 cases (15 attacks) treated with IVIg as a rescue therapy have been reported in the literature and IVIg was efficacious in 10 attacks (Table 1 (b)).^2–7^ The main reason why IVIg was administered in those cases was unresponsiveness to IVMP or PLEX. In some patients unresponsive to IVIg, the median time from relapse to IVIg was longer than the intervals in those who responded to IVIg (3.5 months vs a week), suggesting early institution of IVIg is associated with better outcomes.^7^ Owing to clinical deterioration during IVMP, IVIg was started without delay in our patient, which probably contributed to a favorable therapeutic response although a delayed effect of IVMP cannot be completely ruled out. Given that many women with seropositive NMO are of childbearing age, IVIg may be useful as rescue therapy in AQP4-IgG-positive NMO attacks refractory to IVMP in pregnant patients, especially when complicated with infectious diseases.
